# Targeting Insulin Resistance and Liver Fibrosis: CKD Screening Priorities in MASLD

**DOI:** 10.3390/biomedicines13040842

**Published:** 2025-04-01

**Authors:** Tianyuan Yang, Bingqing Yang, Jingya Yin, Chenxue Hou, Qi Wang

**Affiliations:** 1 Center of Liver Diseases, Beijing Ditan Hospital, Capital Medical University, Beijing 100015, China; tyy66303@gmail.com (T.Y.); ybq1593572022@163.com (B.Y.); hyxiaoya@163.com (J.Y.); 2 Beijing Key Laboratory of Emerging Infectious Diseases, Institute of Infectious Diseases, Beijing Ditan Hospital, Capital Medical University, Beijing 100015, China; 3 Department of Pathology, Beijing Ditan Hospital, Capital Medical University, Beijing 100015, China; hcx20010123@163.com

**Keywords:** MASLD, chronic kidney disease, diabetes, insulin resistance, advanced fibrosis

## Abstract

**Background and Aims:** Chronic kidney disease (CKD) is a recognized extra-hepatic disease of nonalcoholic fatty liver disease (NAFLD). With the redefinition of NAFLD as metabolic dysfunction-associated steatotic liver disease (MASLD), the importance of cardiovascular metabolic factors in MASLD has been highlighted. However, whether MASLD remains independently associated with the prevalence of CKD is yet to be determined. **Method:** We analyzed data from 6567 non-pregnant adults from the National Health and Nutrition Examination Survey 2017–2020. MASLD was identified using liver ultrasound transient elastography and five cardiovascular risk factors. Multivariate logistic regression, subgroup analysis, and restricted cubic splines were employed to explore the associations and interactions within the data. **Results:** The prevalence of CKD across MASLD subgroups with different combinations of cardiometabolic risk factors varied. Univariate regression analysis indicated a significant association between MASLD and CKD (OR: 1.68, *p* < 0.001). This association was not significant after adjusting for diabetes (OR: 0.94, *p* = 0.74) or insulin resistance (OR: 1.00, *p* = 0.98) and was not significant in the fully adjusted model (OR: 0.87, *p* = 0.64). Subgroup analysis confirmed insulin resistance as a modifier in the MASLD-CKD relationship (*p* for interaction = 0.02). Multivariate analysis revealed that liver stiffness measurements (LSMs) were independently associated with CKD. LSM values showed an S-shaped correlation with CKD, with risk increasing above the 8.612 kPa threshold. **Conclusions:** This study suggests that the direct relationship between MASLD and CKD diminished when accounting for insulin resistance. Nevertheless, liver fibrosis emerges as an independent CKD risk factor, emphasizing the critical need for targeted CKD screening among MASLD patients, particularly those with insulin resistance or advanced fibrosis.

## 1. Introduction

The global prevalence of non-alcoholic fatty liver disease (NAFLD) is increasing, positioning it as the predominant chronic liver disorder in the present era [1]. NAFLD not only leads to liver-related mortality, primarily due to cirrhosis and hepatocellular carcinoma [2], but also has a significant impact on various extra-hepatic chronic conditions, exacerbating health issues beyond the liver [3]. It is often associated with adverse extra-hepatic outcomes such as cardiovascular diseases [4], type 2 diabetes [5], chronic kidney disease (CKD) [6], and cholelithiasis [7].

CKD is a notable extra-hepatic disorder that often results from hypertension and diabetes, while also being frequently associated with other conditions such as NAFLD, obesity, and metabolic syndrome [6,8], but the exact relationship between NAFLD and CKD is partly controversial. On one hand, research indicates a higher prevalence and incidence of CKD among individuals with NAFLD [9,10]. Epidemiological data suggest that NAFLD could act as an independent risk factor for CKD [11]. However, the contributing factors of metabolic syndrome also have a partially causal role in the onset of CKD [8], suggesting that the link between NAFLD and CKD might be intricately connected to metabolic syndrome rather than being entirely independent. Recent evidence from the UK Biobank cohort highlights that individual metabolic syndrome traits and their cumulative burden significantly amplify CKD risk in MASLD [12]. In fact, cohort studies on NAFLD and CKD also exhibit flaws such as selection bias [13], overgeneralization inferences [14], and the application of NAFLD diagnostic methods with limited accuracy [15], leading to a wide variability in research findings. On the other hand, there is presently an absence of conclusive evidence to confirm NAFLD as an independent risk factor for CKD on a biological research level.

Following the renaming of NAFLD to metabolic dysfunction-associated steatotic liver disease (MASLD) [16], there is a heightened focus on the influence of cardiometabolic risk factors in MASLD’s advancement [17]. The multi-society redefinition of NAFLD as MASLD, which includes five cardiometabolic risk factors in its diagnostic criteria, could potentially add complexity to its association with CKD. From this perspective, the relationship between MASLD, with its associated cardiometabolic risk factors, and CKD is becoming increasingly intricate, necessitating further exploration.

In our research, we employed data from a United States community cohort, the National Health and Nutrition Examination Survey (NHANES), from 2017 to March 2020. The aim was to explore the relationship between the newly defined MASLD and the prevalence of CKD, offering valuable perspectives on screening for extra-hepatic complications within the MASLD demographic.

## 2. Methods

### 2.1. Study Design

NHANES, managed by the National Center for Health Statistics (NCHS), assesses U.S. health and nutrition through a stratified multistage approach. To counterbalance the impact of the COVID-19 pandemic on the 2019–2020 data, these were combined with the 2017–2018 data to ensure national representativeness. Detailed methodologies of data collection are outlined in the referenced literature [18]. The initial NHANES protocol received approval from the Research Ethics Review Board of the Centers for Disease Control and Prevention, with written informed consent obtained from all adult participants. Human research participants have been recruited in accordance with the Declaration of Helsinki. This study followed the Strengthening the Reporting of Observational Studies in Epidemiology (STROBE) reporting guideline for cohort studies [19]. As a cross-sectional design, this study collects data at a single point in time, allowing for the assessment of associations between variables but not for the determination of causality.

### 2.2. Data Collection

**Figure 1:**
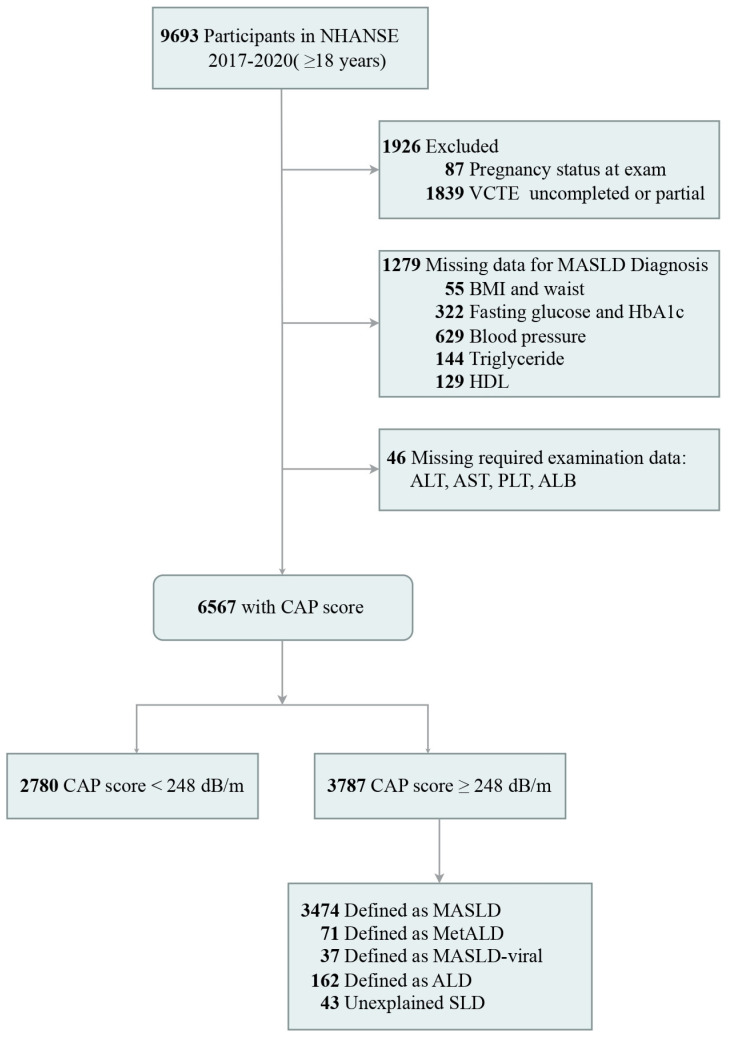
Flow of participants through the study.

Our study encompassed 9606 non-pregnant adult participants from the NHANES 2017–2020. We excluded 1839 individuals due to the absence of diagnostic data for MASLD, including liver ultrasound transient elastography, body mass index (BMI), waist circumference, blood pressure, triglyceride levels, direct high-density lipoprotein cholesterol (HDL-C), and hemoglobin A1c (HbA1c). This resulted in a final analytic cohort of 6567 participants, as detailed in Figure 1.

In NHANES 2017–2020, participants were randomly selected within each age group (age ≥ 12 years) with the same sampling probabilities, which resulted in 3287 participants lacking HOMA-IR data. Those selected were instructed to fast for a duration ranging from 8 to 23 h. Participants who adhered to the fasting requirements then underwent fasting blood sampling. We compared the baseline characteristics between the overall population and those with HOMA-IR data, as detailed in the Table S1.

The homeostasis model assessment-estimated insulin resistance (HOMA-IR score) was calculated using the formula: [fasting insulin (mU/mL) × fasting glucose (mmol/L)/22.5], with insulin resistance designated as a HOMA-IR value of 2.69 or greater [20].

### 2.3. Diagnostic Criteria of NAFLD and MASLD

Fibroscan with Controlled Attenuation Parameter (CAP) was used to identify steatotic liver disease (SLD), with a CAP of ≥248 dB/m indicating mild hepatic steatosis, as evidenced in prior research [21]. Metabolic dysfunction in SLD was diagnosed based on one or more cardiometabolic risk factors [16], detailed criteria are provided in the Supplementary Methods. In SLD, ALD was identified in individuals consuming > 60 g (men) or >50 g (women) of alcohol daily. MASLD was diagnosed if cardiometabolic risks were present with alcohol intake < 30 g (men) or <20 g (women) per day; MetALD for intake between 30 and 60 g (men) and 20–50 g (women). MASLD-Viral hepatitis was noted for co-occurrence with viral hepatitis. Unexplained SLD was classified without metabolic dysfunction, significant alcohol, or viral hepatitis.

NAFLD was defined by evidence of hepatic steatosis with a CAP ≥ 248 dB/m and the exclusion of significant alcohol consumption (defined as daily intake ≥ 20 g for men and ≥ 10 g for women) and other competing causes for hepatic steatosis (positive for the Hepatitis B surface antigen or antibodies indicative of a Hepatitis C infection).

### 2.4. Assessment of Chronic Kidney Disease

The Chronic Kidney Disease Epidemiology Collaboration (CKD-EPI) equation was utilized to calculate the estimated glomerular filtration rate (eGFR) [22], detailed in the Supplementary Methods, and the staging of CKD follows the Kidney Disease: Improving Global Outcomes (KDIGO) 2024 guidelines [23], with stages outlined in the Supplementary Methods.

### 2.5. Statistical Analysis

All analyses incorporated the NHANES sample weights and accounted for the complex sample survey design using standard methods. Weighted prevalence estimates were calculated by applying the provided sampling weights, stratification variables, and clustering units to generate nationally representative proportions. The comparison of baseline characteristics was conducted using the *t*-test or Mann–Whitney U test for continuous variables and the design-adjusted Rao–Scott chi-squared test for categorical variables. To examine the relationship between selected demographic and cardiometabolic risk factors, MASLD, and CKD, weighted univariate and multivariate logistic regression analyses were utilized. Additionally, to probe into the potential interactive effects between variables on CKD, interaction terms were incorporated into the weighted logistic regression. In subgroup analyses, interactions on the multiplicative scale were evaluated by conducting likelihood ratio tests. The associations between CKD and liver stiffness measurement (LSM) values were assessed on a continuous scale with restricted cubic spline curves based on logistic models. Knot selection was based on the Akaike Information Criterion (AIC). All statistical procedures were conducted using R software, version 4.3.2. A two-tailed *p*-value of less than 0.05 was considered statistically significant.

## 3. Results

### 3.1. Baseline Characteristics of Participants

Table 1 details the baseline characteristics of the participants, categorized by the diagnosis of MASLD and NAFLD. Regarding renal function, the MASLD group exhibited a higher prevalence of CKD (17.52%) compared to the non-MASLD group (11.06%, *p* < 0.001). Additionally, the MASLD group had a lower eGFR (91.47 vs. 96.38 mL/min/1.73 m^2^, *p* < 0.001), higher serum creatinine levels (0.88 vs. 0.87 mg/dL, *p* = 0.018), and a higher albumin-to-creatinine ratio (ACR) (7.78 vs. 6.75 mg/g, *p* = 0.002) compared to the non-MASLD group. These renal function trends were also mirrored in the NAFLD group.

### 3.2. CKD Weighted Prevalence in MASLD with Combinations of Five Cardiometabolic Risk Factors

**Figure 2:**
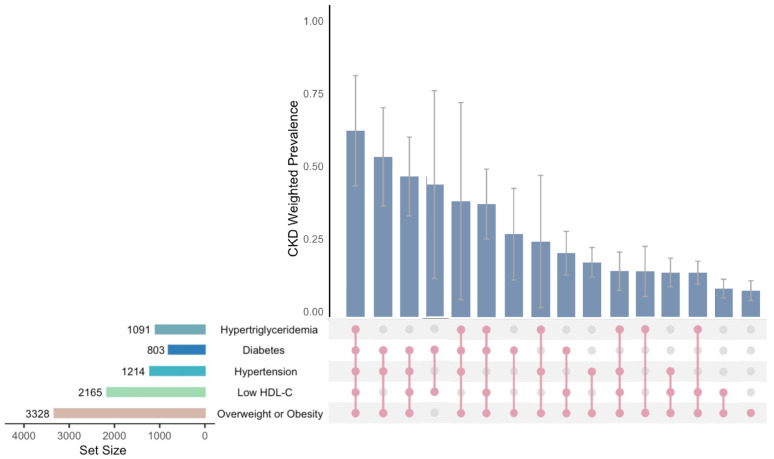
Weighted prevalence of CKD in MASLD different combinations of five cardiometabolic risk factors.

We examined 31 subgroups within the MASLD cohort, categorized based on a combination of five cardiometabolic risk factors. The weighted prevalence of CKD across these subgroups is shown in Table 2 and Figure 2. Subgroups with fewer participants (highlighted in Table 2) are temporarily excluded from this discussion.

Among the 16 subgroups included in the discussion, in the seven subgroups with higher prevalence rates (Subgroup 1–7), all patients with MASLD also had diabetes.

Further analysis indicates that the subgroup of MASLD patients with all five cardiometabolic risk factors (Subgroup 1) showed the highest CKD prevalence at 64.0% (95% CI, 45.0–83.0%). There was a noticeable decrease in CKD prevalence among patients with four risk factors, excluding hypertension (Subgroup 6), compared to Subgroup 1 (64.0% vs. 38.8%, *p* = 0.03). Individuals diagnosed with overweight or obesity alone (Subgroup 16) had a lower CKD prevalence of 8.9% (95% CI, 5.5–12.3%), with no significant difference observed when this condition was paired with low HDL-C (Subgroup 16 vs. Subgroup 15: 8.9% vs. 9.7%, *p* = 0.73), or with hypertriglyceridemia (Subgroup 16 vs. Subgroup 12: 8.9% vs. 15.5%, *p* = 0.16).

### 3.3. Association of MASLD and Cardiometabolic Risk Factors with CKD

To explore the interrelationships between MASLD, cardiometabolic risk factors, and the prevalence of CKD, we conducted weighted univariate logistic regression analyses and multifactorial regression analyses (Table 3). Detailed information on other variables is presented in Table S2.

Univariate regression analysis revealed that MASLD is significantly linked to the prevalence of GFR category ≥ G1 (OR: 1.68, 95% CI: 1.38–2.04; *p* < 0.001) and GFR category ≥ G3 (OR: 1.84, 95% CI: 1.36–2.48; *p* = 0.001). Each cardiometabolic risk factor was associated with an increased risk of CKD, with diabetes being the most impactful, amplifying the risk for GFR category ≥ G1 and ≥G3 by 5.37 (95% CI: 4.53–6.35; *p* < 0.001) and 3.68 (95% CI: 2.87–4.72; *p* < 0.001) times, respectively.

Upon adjusting for age and sex, MASLD maintained its significant link to GFR category ≥ G1 (OR: 1.32, 95% CI: 1.07–1.61; *p* = 0.01).

In Models 1 through 5, which adjusted for age, sex, and each cardiometabolic risk factor individually, MASLD’s association with GFR category ≥ G1 persisted, except when adjustments were made for low HDL-C (Model 4: OR: 1.15, 95% CI: 0.93–1.42; *p* = 0.20) and diabetes (Model 5: OR: 1.06, 95% CI: 0.86–1.31; *p* = 0.54). For GFR category ≥ G3, the associations were not significant after adjusting for any cardiometabolic risk factors.

Model 6, which adjusted for age, sex, hypertension, overweight/obesity, hypertriglyceridemia, and low HDL-C, retained a significant association for GFR category ≥ G1 (OR: 1.32, 95% CI: 1.03–1.69; *p* = 0.03). However, in Model 7, which additionally adjusted for diabetes based on Model 6, the association between MASLD and GFR category ≥ G1 was statistically insignificant (OR: 0.94, 95% CI: 0.65–1.47; *p* = 0.74). For GFR category ≥ G3, neither Model 6 nor Model 7 showed insignificant associations.

The fully adjusted model (Model 8), which accounted for age, sex, all cardiometabolic risk factors, smoking, alcohol consumption, and antihypertensive medication use, found no significant association between MASLD and GFR category ≥ G1 (OR: 0.87, 95% CI: 0.46–1.63; *p* = 0.64). or ≥G3 (OR: 0.85, 95% CI: 0.40–1.82; *p* = 0.65).

### 3.4. The Effect of Insulin Resistance on the Relationship Between MASLD and CKD

In Table 4, when insulin resistance was introduced as a categorical variable into the adjusted weighted regression model (Model 9), the association between MASLD and CKD remained insignificant (OR: 1.00, 95% CI: 0.73–1.38; *p* = 0.98). Considering previous research suggesting that insulin resistance as a potential pathophysiological mechanism through which NAFLD may precipitate the onset of CKD [6], the introduction of an interaction term between insulin resistance and MASLD into the model was statistically significant (OR: 2.02, 95% CI: 1.05–3.89; *p* = 0.04). Our exploratory analysis suggests a potential modifying role of insulin resistance in the MASLD–CKD association, though this finding did not reach statistical significance after adjustment for multiple comparisons (adjusted *p* = 0.09).

### 3.5. Subgroup and Interaction Analysis

**Figure 3:**
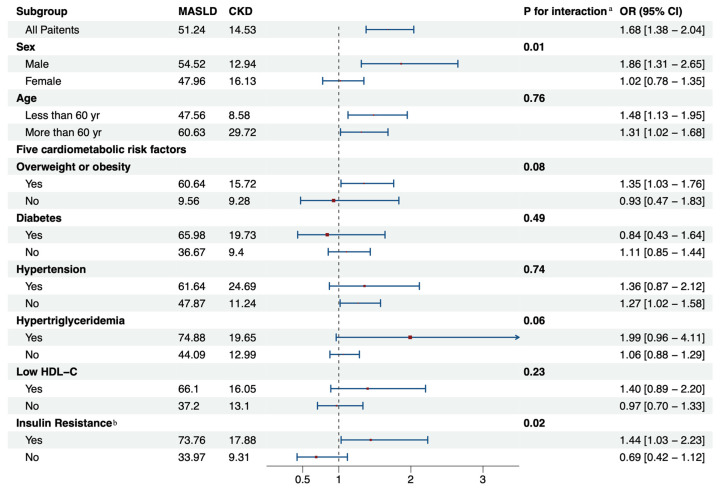
Association of MASLD with CKD in predefined subgroups. Adjusted for all factors (age, sex, all cardiometabolic risk factors, smoking, alcohol consumption, and antihypertensive medication use) except the subgroup factor itself. ^a^ Diabetes adjustments are excluded in the insulin resistance subgroup. ^b^ interactions on the multiplicative scale were evaluated by conducting likelihood ratio tests. Abbreviations: OR, odds ratios; CI, confidence intervals; CKD, chronic kidney disease; MASLD, metabolic dysfunction-associated steatotic liver disease; HDL-C, lipoprotein cholesterol.

The relationship between MASLD and CKD demonstrated variability between different subgroups (Figure 3). The analyses identified gender (*p_for interaction_* = 0.01) and insulin resistance (*p_for interaction_* = 0.02) as potential modifiers of the MASLD–CKD association. In individuals without insulin resistance, the influence of MASLD on CKD risk is not notable (OR: 0.69, 95% CI: 0.42–1.12; *p* = 0.12). Conversely, for those with insulin resistance, MASLD significantly increases CKD risk (OR: 1.44, 95% CI: 1.03–2.23; *p* = 0.05). Moreover, in the subgroup analysis focusing on the presence of diabetes, the association between MASLD and CKD remained non-significant, whether diabetes was present (OR: 0.84, 95% CI: 0.43–1.64; *p* = 0.59) or absent (OR: 1.11, 95% CI: 0.85–1.44; *p* = 0.43).

### 3.6. Association of Liver Fibrosis and CKD

**Figure 4:**
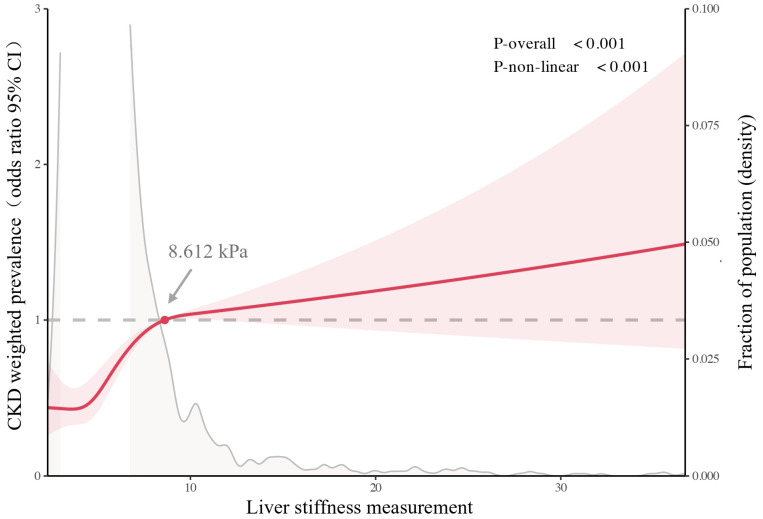
Multivariable-adjusted odds ratios for CKD prevalence according to LSM values on a continuous scale. The solid red line delineates the multivariable-adjusted odds ratios, with the red shading indicating the 95% confidence intervals, as determined by restricted cubic spline regressions with four knots. The grey dashed line marks the reference point with an odds ratio of 1.0. Grey solid lines depict the distribution of the population across LSM levels, with parts not fully displayed due to larger proportions. Arrows point to the inflection points of the curve where the OR reaches 1, notably at an LSM value of 8.612 kPa. The analysis was adjusted for age, sex, all cardiometabolic risk factors, smoking, alcohol consumption, and antihypertensive medication use.

Our study revealed that liver fibrosis, assessed using LSM, is independently associated with an elevated risk of CKD in patients with MASLD. Multivariable logistic regression confirmed a significant increase in CKD risk for LSM ≥ 8.612 kPa (OR = 2.72, 95% CI:1.30–5.68, *p* = 0.01). Restricted cubic spline analysis further demonstrated a nonlinear relationship, with CKD risk rising sharply beyond this threshold (Figure 4). Notably, the threshold of 8.612 kPa closely aligns with the established cutoff for advanced liver fibrosis (LSM ≥ 8 kPa). Given the clinical practicality of transient elastography, we propose LSM ≥ 8 kPa as a screening benchmark to identify MASLD patients at high CKD risk. Individuals meeting this threshold should undergo confirmatory renal function tests to guide early intervention. However, the widening confidence intervals at higher LSM values may reflect the limited sample size in this subgroup, and further validation is warranted.

## 4. Discussion

In our cross-sectional analysis of NHANES 2017–2020 data, we noted a significantly higher weighted CKD prevalence in MASLD individuals with diabetes versus those without. We did not find an independent association between MASLD and CKD. Insulin resistance may modify the MASLD-CKD association (interaction *p* = 0.04; FDR-adjusted *p* = 0.09), though this finding requires cautious interpretation due to its exploratory nature. Moreover, liver fibrosis remained associated with CKD in the fully adjusted model. An increase in LSM values beyond a certain threshold became a potential risk factor for CKD.

From epidemiological and pathophysiological perspectives, diabetes emerges as an unavoidable pivotal topic in the clinical diagnosis and treatment of MASLD. Globally, the prevalence of MASLD among individuals with T2DM is as high as 67%, with projections suggesting that by 2045, there will be 469 million individuals worldwide with concurrent MASLD and T2DM [24,25]. Furthermore, MASLD and diabetes share numerous overlapping physiological processes, with studies confirming a bidirectional and synergistic relationship between MASLD and diabetes [26]. The rebranding of NAFLD to MASLD further underscores the critical link between MASLD and cardiometabolic risk factors.

Prior studies have also questioned the independent association between NAFLD and CKD. Jeffrey and colleagues’ cross-sectional study of 11,469 adults using data from NHANES III (1988–1994) [27], finding an association between NAFLD and Stage 3 or higher CKD weakened after adjusting for demographic data and components of metabolic syndrome (OR: 1.04, 95% CI: 0.88–1.23, *p* = 0.64). In another study, similarly, Robert et al., using CT scans to measure hepatic fat in 987 participants from the Framingham Heart Study cohort [28], found no significant link between liver fat and CKD, microalbuminuria, or eGFR. They suggested that the association between NAFLD and CKD could be explained by shared risk factors, without specifying which factors had the most significant impact. Compared to previous studies, our research has a clear advantage. Our study extends this evidence by leveraging the NHANES 2017–2020 dataset, which incorporates liver ultrasound transient elastography—a more sensitive tool for diagnosing hepatic steatosis and fibrosis—to redefine MASLD under current criteria. Importantly, we shift the focus from broad metabolic syndrome to insulin resistance. Although the interaction between MASLD and insulin resistance showed only borderline significance after multiple testing correction (adjusted *p* = 0.09), the direction and magnitude of the effect (OR = 2.02) align with mechanistic hypotheses positing insulin resistance as a shared driver of both MASLD progression and renal injury. This suggests that targeted management of insulin resistance in MASLD patients may mitigate CKD risk—a hypothesis warranting validation in longitudinal studies.

Indeed, our findings present a challenge to some earlier conclusions. Previous cross-sectional and longitudinal studies have shown associations between NAFLD and CKD, which contrasts with our results. There are several potential explanations for this difference. First, some of these studies were hospital-based and thus might have been subject to referral bias [13], selecting patients with more severe liver conditions. Second, regarding inclusion and exclusion criteria in cohort studies, some excluded diabetic patients [14] from the outset or exclusively involved diabetic populations [29], and not all studies covered adults across all age groups [14]. Statistical analyses conducted in cohorts with specific characteristics, then generalized to all NAFLD populations to suggest an independent association between NAFLD and CKD, may be overly broad in their conclusions. Third, some studies did not adjust for covariates such as diabetes [13] or antihypertensive medication use [30,31]. Lastly, Sun et al.’s study on the association between MAFLD and CKD using NHANES III data did not employ weighted analyses in their statistical strategy [32].

When two prevalent diseases coexist and share common risk factors, disentangling their causal relationship and understanding the role of potential confounders can be quite challenging. Emerging evidence suggests that the association between MASLD and CKD may primarily stem from metabolic dysfunction. A 10-year cohort study (n = 12,138) found that MASLD and alcohol-associated liver disease, but not non-metabolic SLD were linked to CKD risk [33]. This contrast may indicate that metabolic abnormalities, rather than hepatic steatosis itself, could be the key drivers of renal injury in MASLD. Our study, being cross-sectional, cannot conclude causality between the two conditions. However, in multifactorial regression analyses, the diminished association between MASLD and CKD after controlling for insulin resistance suggests that the relationship between these diseases may be confounded by shared pathophysiological mechanisms and common cardiometabolic risk factors. In fact, peripheral studies can provide valuable insights. In subjects with central obesity and T2DM, insulin resistance often co-occurs with other cardiometabolic risk factors that increase the risk for NAFLD and CKD [34]. An influential review by Christopher et al. also highlighted that NAFLD, CKD, and T2DM/Metabolic Syndrome share many traditional risk factors [6]. With ongoing research, emerging risk factors frequently coexist with these conditions, including perturbations of the intestinal microbiota (dysbiosis) with associated inflammation [35,36] and platelet activation [37,38].

Our analysis also explored the increased risk of CKD associated with liver fibrosis, aligning with findings from previous research. Recent studies have highlighted that liver fibrosis synergistically increases CKD risk in MASLD [12,39] and that MASLD severity predicts CKD progression after remission [40]. Our study further identified a non-linear relationship between LSM values and the risk of CKD. Once the LSM values exceeded 8.612 kPa, they became a potential risk factor for CKD, with the risk escalating as LSM values increased. This threshold is close to the current criterion using LSM values to define advanced fibrosis (LSM ≥ 8 kPa) [41], suggesting that employing LSM ≥ 8 kPa as a screening threshold for CKD may be appropriate or warrant discussion. The widening confidence intervals at LSM values ≥ 10 kPa (Figure 4) likely reflect reduced statistical power in this subgroup, as fewer patients exhibited such advanced fibrosis in our cohort. Future studies with larger samples of high-LSM patients are needed to clarify risk patterns in severe fibrosis.

Our study is subject to the following limitations: First, due to its cross-sectional nature, it cannot establish a causal relationship between MASLD and CKD. Second, potential selection bias may arise from the exclusion of missing values, given that some data were incomplete within the NHANES database. Third, considering that our study is derived from a U.S. cohort, the extrapolation of results may be influenced by the sample origin. Lastly, our study utilized liver ultrasound transient elastography to diagnose MASLD and liver fibrosis, which, while less accurate than the gold standard of liver biopsy, is still a practical choice due to considerations of feasibility in large-scale, national community cohort studies. Despite this, liver ultrasound transient elastography maintains higher accuracy compared to other non-invasive diagnostic methods.

## 5. Conclusions

Our study highlights insulin resistance as a key factor in the MASLD–CKD relationship. The association between this relationship is significantly influenced by the presence of diabetes and insulin resistance. Given the current global constraints on healthcare resources, it is important to allocate medical resources judiciously while promptly identifying high-risk individuals with concurrent MASLD and CKD. Based on our findings, it is advisable to closely monitor renal function in patients with MASLD coexisting with diabetes. For MASLD patients without diabetes, assessments for insulin resistance can identify those at high risk of CKD. In the non-cirrhotic stages of MASLD, managing diabetes may reduce the probability of developing CKD. Moreover, not only is liver fibrosis identified as an independent risk factor for CKD, but the utility of LSM values ≥ 8 kPa also merits further investigation for stratifying CKD risk in MASLD patients, extending its utility beyond its conventional role in diagnosing advanced fibrosis.

## Figures and Tables

**Table 1:** Baseline characteristics in individuals with and without MASLD and in those with and without NAFLD ^a^.

Variables	Whole Cohort (n = 6567)		MASLD (n = 3474)		Non-MASLD (n = 3093)		*p*-Value ^b^	NAFLD (n = 3333)		Non-NAFLD (n = 3234)		*p*-Value
Demographics												
Age, y	47.27	±17.41	50.71	±16.46	43.66	±17.66	<0.001	50.76	±16.57	44.01	±17.56	<0.001
Sex							0.002					0.007
Male, %	3298	(50.07)	1812	(53.27)	1486	(46.71)		1736	(53.28)	1562	(47.07)	
Female, %	3269	(49.93)	1662	(46.73)	1607	(53.29)		1597	(46.72)	1672	(52.93)	
Five cardiometabolic risk factors												
Overweight or Obesity, %	5342	(81.61)	3328	(96.57)	2014	(65.88)	<0.001	3192	(95.32)	2150	(68.76)	<0.001
BMI, kg/m^2^	29.47	±6.89	32.72	±6.84	26.05	±5.05	<0.001	32.70	±6.98	26.45	±5.26	<0.001
Waist circumference, cm	99.92	±16.62	108.57	±14.88	90.85	±13.16	<0.001	108.35	±15.26	92.06	±13.74	<0.001
Hypertension, %	1968	(24.50)	1214	(29.47)	754	(19.28)	<0.001	1165	(29.47)	803	(19.85)	<0.001
Diastolic blood pressure, mmHg	73.83	±10.74	75.72	±10.61	71.84	±10.51	<0.001	75.56	± 10.71	72.2	± 10.50	<0.001
Systolic blood pressure, mmHg	121.52	±16.97	123.67	±16.70	119.26	±16.95	<0.001	123.61	±16.80	119.57	±16.90	<0.001
Diabetes, %	1016	(11.48)	803	(18.39)	213	(4.22)	<0.001	764	(19.01)	252	(44.35)	<0.001
Hypertriglyceridemia, %	1460	(23.22)	1091	(33.94)	369	(11.97)	<0.001	1050	(34.46)	410	(12.70)	<0.001
Low HDL-C, %	3241	(48.59)	2165	(62.68)	1076	(33.79)	<0.001	2072	(63.20)	1169	(34.91)	<0.001
Lab panel												
Total cholesterol, mg/dL	187.37	±40.53	189.99	±41.21	184.61	±39.62	0.001	189.41	±41.34	185.45	±39.66	0.03
Triglycerides, mg/dL	138.00	(78.00, 162.00)	164.00	(95.00, 159.70)	110.00	(66.00, 129.00)	<0.001	165.00	(94.00, 159.00)	112.00	(67.00, 132.00)	<0.001
HDL-C, mg/dL	53.62	±15.67	48.94	±13.33	58.53	±16.43	<0.001	48.62	±12.90	58.3	±16.57	<0.001
HbA1c, %	5.64	±0.94	5.88	±1.13	5.40	±0.59	<0.001	5.89	±1.13	5.41	±0.62	<0.001
ALT, U/L	23.00	(13.00, 27.00)	25.00	(15.00, 31.00)	20.00	(12.00, 23.00)	<0.001	24.00	(15.00, 30.00)	21.00	(13.00, 24.00)	<0.001
AST, U/L	21.00	(16.00, 24.00)	21.00	(16.00, 24.00)	21.00	(16.00, 23.00)	0.30	21.00	(16.00, 24.00)	22.00	(16.00, 23.00)	0.20
ALP, U/L	74.96	±24.30	77.79	±23.44	71.98	±24.83	<0.001	78.14	±23.61	71.98	±24.57	<0.001
GGT, U/L	29.00	(14.00, 31.00)	32.00	(16.00, 35.00)	25.00	(12.00, 26.00)	<0.001	31.00	(16.00, 34.00)	26.00	(12.00, 27.00)	<0.001
Albumin, g/L	41.23	±3.22	40.85	±3.17	41.64	±3.22	<0.001	40.80	±3.18	41.64	±3.21	<0.001
Globulin, g/L	29.83	±4.06	30.08	±4.00	29.56	±4.11	0.001	30.15	±4.00	29.53	±4.09	0.001
Platelet, 10^9^/L	246.60	±62.40	250.05	±63.75	242.97	±60.74	0.03	249.99	±63.72	243.42	±60.97	0.05
Total bilirubin, umol/L	8.11	(5.13, 10.26)	7.98	(5.13, 8.55)	8.24	(5.13, 10.26)	0.20	7.95	(5.13, 8.55)	8.26	(5.13, 10.26)	0.20
eGFR, mL/min/1.73 m^2^	93.86	±21.34	91.47	±21.36	96.38	±21.03	<0.001	91.39	±21.55	96.17	±20.89	<0.001
Creatinine, mg/dL	0.88	±0.34	0.88	±0.38	0.87	±0.28	0.02	0.89	±0.39	0.87	±0.28	0.04
Urinary ACR, mg/g	7.27	(4.73, 13.81)	7.78	(5.00, 15.32)	6.75	(4.51, 12.19)	0.002	7.84	(5.00, 15.62)	6.74	(4.51, 12.18)	0.003
Noninvasive tests												
Steatosis												
CAP, dB/m	263.77	±62.31	308.32	±40.74	216.93	±44.04	<0.001	307.89	±40.99	222.45	±49.14	<0.001
CAP ≥ 248 dB/m, %	3787	(56.97)	3474	(100)	313	(11.75)	<0.001	3333	(100)	454	(16.68)	<0.001
Fibrosis												
LSM, kPa	5.70	(4.10, 6.10)	6.30	(4.30, 6.60)	5.00	(3.80, 5.60)	<0.001	6.40	(4.30, 6.60)	5.00	(3.80, 5.60)	<0.001
LSM ≥ 8 kPa, %	660	(9.08)	498	(13.89)	162	(4.03)	<0.001	476	(14.24)	184	(4.27)	<0.001
GFR category												
G1, %	318	(4.84)	204	(5.87)	114	(3.69)	<0.001	198	(5.94)	120	(3.71)	<0.001
G2, %	281	(4.29)	180	(5.18)	101	(3.26)	<0.001	175	(5.25)	106	(3.27)	<0.001
≥G3, %	352	(5.36)	225	(6.47)	127	(4.11)	<0.001	219	(6.57)	133	(4.10)	<0.001
	(n = 3280) ^c^		(n = 1728)		(n = 1552)			(n = 1657)		(n = 1623)		
Insulin resistance, %	1594	(44.62)	1184	(63.63)	410	(24.26)	<0.001	1138	(64.23)	456	(26.18)	<0.001
HOMA-IR	3.95	(1.54, 4.61)	5.53	(2.38, 6.22)	2.25	(1.40, 2.76)	<0.001	5.56	(2.39, 6.21)	2.43	(1.17, 2.88)	<0.001

Note: National Health and Nutrition Examination Surveys cycle 2017–2020, n = 6567. MASLD, metabolic dysfunction-associated steatotic liver disease; NAFLD, non-alcoholic fatty liver disease; BMI, body mass index; HDL, high-density lipoprotein; HbA1c, glycosylated hemoglobin; ALT, alanine aminotransferase; AST, aspartate aminotransferase; ALP, Alkaline phosphatase; GGT, gamma-glutamyl transferase; eGFR, estimated glomerular filtration rate; ACR, albumin-to-creatinine ratio; CAP, controlled attenuation parameter; LSM, liver stiffness measure; HOMA-IR: homeostasis model assessment-estimated insulin resistance. ^a^ Method: For categorical variables: “count (weighted percentage)”; for continuous variables with normal distribution: “mean ± standard deviation”; for continuous variables with non-normal distribution: “median (interquartile range)”. ^b^ Continuous variables were compared using *t*-tests and Mann–Whitney U tests, and categorical variables using the Rao–Scott chi-squared tests. ^c^ Due to the random selection of participants for fasting blood sampling in each age group, only 3280 individuals in the study population had computable HOMA-IR values.

**Table 2:** Weighted prevalence of CKD in MASLD different combinations of five cardiometabolic risk factors.

Subgroup	Overweight or Obesity	Low HDL-C	Hypertension	Diabetes	Hypertrigly-Ceridemia	CKD (n = 752)	Non-CKD (n = 2722)	MASLD Weighted Prevalence (95% CI)	
1	√	√	√	√	√	66	49	64.0%	(45.0–83.0%)
2	√		√	√		44	41	55.0%	(38.0–71.9%)
3	√	√	√	√		60	65	48.2%	(34.6–61.8%)
4		√		√		2	5	45.1%	(12.9–77.4%)
5	√		√	√	√	9	7	39.9%	(5.9–73.8%)
6	√	√		√	√	72	94	38.8%	(26.8–50.9%)
7	√			√		27	75	28.4%	(12.6–44.2%)
8	√		√		√	11	30	25.9%	(3.2–48.7%)
9	√	√		√		39	104	22.0%	(14.5–29.4%)
10	√		√			73	220	18.8%	(13.7–23.9%)
11	√	√	√		√	39	175	15.6%	(9.0–22.2%)
12	√				√	13	63	15.5%	(6.9–24.1%)
13	√	√	√			59	210	15.2%	(10.2–20.1%)
14	√	√			√	61	335	15.1%	(11.2–19.0%)
15	√	√				74	586	9.7%	(6.5–13.0%)
16	√					70	527	8.9%	(5.5–12.3%)
17 ^a^			√			7	17	15.4%	(0.0–30.7%)
18	√			√	√	7	23	18.1%	(0.0–36.7%)
19		√				5	19	13.8%	(0.0–30.3%)
20		√	√			3	13	18.3%	(0.0–41.8%)
21		√	√		√	2	6	20.7%	(0.0–46.2%)
22		√	√	√		2	0	100.0%	—
23				√	√	1	0	100.0%	—
24		√	√	√	√	1	1	5.1%	(0.7–4.3%)
25 ^b^						5	28	11.5%	(0.0–23.7%)
26		√			√	0	13	—	
27		√		√	√	0	5	—	
28					√	0	4	—	
29				√		0	3	—	
30			√		√	0	3	—	
31			√	√	√	0	1	—	

MASLD, metabolic dysfunction-associated steatotic liver disease; CKD, chronic kidney disease; HDL, high-density lipoprotein; CI, confidence interval. ^a^: Due to the small sample sizes, the confidence intervals within the shaded areas are unreliable, and thus, these data are excluded from the discussion. ^b^: Given the broader criteria for elevated glucose in MASLD diagnosis compared to diabetes, some individuals may have elevated glucose without diabetes or other cardiometabolic risk factors.

**Table 3:** Weighted logistic regression analyses of the association between variables and CKD Prevalence.

		GFR Category ≥ G1			GFR Category ≥ G3		
Model	Variables	OR (95% CI)		*p*-Value	OR (95% CI)		*p*-Value
Unadjusted model							
	No MASLD	Reference			Reference		
	MASLD	1.68	(1.38–2.04)	<0.001	1.84	(1.36–2.48)	0.001
	No Overweight or Obesity	Reference			Reference		
	Overweight or Obesity	1.82	(1.36–2.44)	0.001	3.12	(2.15–4.53)	<0.001
	No Hypertension	Reference			Reference		
	Hypertension	2.59	(2.11–3.17)	<0.001	2.12	(1.61–2.79)	<0.001
	No Diabetes	Reference			Reference		
	Diabetes	5.37	(4.53–6.35)	<0.001	3.68	(2.87–4.72)	<0.001
	No Low HDL-C	Reference			Reference		
	Low HDL-C	1.27	(1.07–1.51)	0.01	1.17	(0.93–1.48)	0.20
	No Hypertriglyceridemia	Reference			Reference		
	Hypertriglyceridemia	1.64	(1.30–1.06)	<0.001	1.67	(1.31–2.15)	<0.001
	Age, y	1.05	(1.05–1.06)	<0.001	1.12	(1.10–1.14)	<0.001
	Sex (Male = 1, Female = 2)	1.29	(1.05–1.60)	0.03	1.26	(1.06–1.50)	0.02
	Systolic blood pressure, mmHg	1.03	(1.02–1.03)	<0.001	1.03	(1.02–1.04)	<0.001
	Diastolic blood pressure, mmHg	1.01	(0.99–1.01)	0.32	0.97	(0.96–0.99)	0.001
	BMI, kg/m^2^	1.03	(1.02–1.04)	<0.001	1.02	(1.01–1.03)	0.001
	Waist circumference, cm	1.02	(1.01–1.03)	<0.001	1.02	(1.02–1.03)	<0.001
	HbA1c, %	1.85	(1.70–2.00)	<0.001	1.37	(1.27–1.48)	<0.001
	CAP, dB/m	1.00	(1.00–1.01)	<0.001	1.00	(1.00–1.01)	0.003
	LSM, kPa	1.04	(1.02–1.06)	<0.001	1.02	(1.01–1.03)	<0.001
Adjustment Model	MASLD						
Age and sex		1.32	(1.07–1.61)	0.01	1.34	(0.99–1.82)	0.06
Model 1		1.29	(1.05–1.59)	0.02	1.34	(0.99–1.32)	0.06
Model 2		1.30	(1.02–1.65)	0.04	1.22	(0.88–1.70)	0.22
Model 3		1.19	(0.98–1.45)	0.07	1.18	(0.86–1.61)	0.29
Model 4		1.15	(0.93–1.42)	0.20	1.15	(0.84–1.58)	0.37
Model 5		1.06	(0.86–1.31)	0.54	1.23	(0.89–1.70)	0.20
Model 6		1.32	(1.03–1.69)	0.03	1.01	(0.72–1.43)	0.94
Model 7		0.94	(0.65–1.47)	0.74	0.97	(0.68–1.39)	0.86
Model 8		0.87	(0.46–1.63)	0.64	0.85	(0.40–1.82)	0.65

MASLD, metabolic dysfunction-associated steatotic liver disease; CKD, chronic kidney disease; BMI, body mass index; HDL, high-density lipoprotein; HbA1c, glycosylated hemoglobin; CAP, controlled attenuation parameter; LSM, liver stiffness measure, CI, confidence interval; OR, odds ratio. Model 1: Adjusted for age, sex. and hypertension. Model 2: Adjusted for age, sex. and overweight or obesity. Model 3: Adjusted for age, sex and hypertriglyceridemia. Model 4: Adjusted for age, sex and Low HDL-C. Model 5: Adjusted for age, sex and Diabetes. Model 6: Adjusted for age, sex, hypertension, overweight or obesity, hypertriglyceridemia, and low HDL-C. Model 7: Further adjusted for diabetes based on Model 6. Model 8 (fully adjusted model): Further adjusted for smoking, alcohol consumption, and antihypertensive medication use based on Model 7.

**Table 4:** Weighted logistic regression on insulin resistance, MASLD, and CKD prevalence.

Model	Variables	OR (95% CI)		*p*-Value	Adjusted-*p*
Unadjusted model					
	No Insulin resistance	Reference			
	Insulin resistance	2.12	(1.74–2.58)	<0.001	
	HOMA-IR	1.06	(1.02–1.09)	0.002	
Adjustment Model					
Model 9	No MASLD	Reference			
	MASLD	1.00	(0.73–1.38)	0.98	
	No Insulin resistance	Reference			
	Insulin resistance	1.91	(1.48–2.46)	<0.001	
Model 9 + Insulin resistance × MASLD ^a^	No MASLD	Reference			
	MASLD	0.71	(0.43–1.16)	0.16	
	No Insulin resistance	Reference			
	Insulin resistance	1.26	(0.77–2.05)	0.33	
	MASLD × Insulin resistance	2.02	(1.05–3.89)	0.04	0.09 ^b^

MASLD, metabolic dysfunction-associated steatotic liver disease; CI, confidence interval; OR, odds ratio. HOMA-IR: homeostasis model assessment-estimated insulin resistance. Model 9: Further adjusted for insulin resistance, based on Model 8, with the exclusion of diabetes. ^a^: Model 9 + Insulin resistance × MASLD: Introduces a multiplicative interaction term between insulin resistance and MASLD in Model 9. ^b^: *p*-values for interaction terms were adjusted using the Benjamini–Hochberg procedure to control the false discovery rate (FDR).

## Data Availability

The datasets generated and analyzed during the current study are available in the National Health and Nutrition Examination Survey (NHANES) repository. The datasets can be accessed at the following persistent web link: https://www.cdc.gov/nchs/nhanes/index.htm, accessed on 24 March 2025.

## Supplementary Materials

The following supporting information can be downloaded at: https://www.mdpi.com/article/10.3390/biomedicines13040842/s1, Table S1. Baseline characteristics of NHANES 2017–2020 individuals with HOMA-IR Data. Table S2. Weighted logistic regression analyses of the association between all variables and CKD Prevalence. Table S3. Interaction Effects of MASLD with Key Variables: Odds Ratios and Confidence Intervals.

## Abbreviations

NAFLD	Non-alcoholic fatty liver disease
CKD	Chronic kidney disease
MASLD	Metabolic dysfunction-associated steatotic liver disease
NHANES	National Health and Nutrition Examination Survey
NCHS	National Center for Health Statistics
BMI	Body mass index
HDL-C	Lipoprotein cholesterol
HbA1c	Hemoglobin a1c
CAP	Controlled attenuation parameter
HOMA-IR	Homeostasis model assessment-estimated insulin resistance
SLD	Steatotic liver disease
ALD	Alcoholic liver disease
MetALD	MASLD with increased alcohol consumption
CKD-EPI	Chronic Kidney Disease Epidemiology Collaboration
eGFR	Estimated glomerular filtration rate
KDIGO	Kidney Disease: Improving Global Outcomes
ACR	Albumin-to-creatinine ratio
LSM	Liver stiffness measurement
OR	Odds ratio
CI	Confidence interval
FLI	Fatty Liver Index
FIB-4	Fibrosis-4 index
