# Supplementary material for: Targeting Insulin Resistance and Liver Fibrosis: CKD Screening Priorities in MASLD
Source: Biomedicines. 2025 Apr 1;13(4):842. doi: 10.3390/biomedicines13040842 (PMC12025161; doi:10.3390/biomedicines13040842)
Supplement: Supplementary file 1 [file biomedicines-13-00842-s001.zip › Supplementary Methods and Results.pdf]

Supplementary Methods

Detailed Calculation Methods for BMI, Diabetes Identification

BMI was derived from weight in kilograms divided by height in meters squared. Diabetes was identified by fasting glucose ≥7.0 mmol/L, HbA1c ≥6.5%, or diabetes medication use.

Criteria for Diagnosing Metabolic Dysfunction in SLD

Metabolic dysfunction in SLD was diagnosed based on one or more cardiometabolic risk factors: ①BMI of 25 kg/m2 or higher, or a waist circumference exceeding 94 cm for males and 80 cm for females, ② Fasting plasma glucose levels of 100 mg/dL or higher, or an HbA1c of 5.7% or above, ③ Blood pressure of 130/85 mmHg or higher, or receiving treatment for hypertension, ④ Triglyceride levels of 150 mg/dL or higher, ⑤ Low HDL-C, defined as less than 40 mg/dL for males and less than 50 mg/dL for females.

Assessment of Chronic Kidney Disease

The Chronic Kidney Disease Epidemiology Collaboration (CKD-EPI) equation was utilized to calculate the estimated glomerular filtration rate (eGFR)[21]. The CKD-EPI equation is detailed as: $eGFR = 141 \times \min(Scr/\kappa,1)^{\alpha} \times \max(Scr/\kappa,1)^{-1.209} \times 0.993^{Age} \times 1.018$ [if female], where Scr is serum creatinine, κ is 0.7 for females and 0.9 for males, α is −0.329 for females and −0.411 for males, min indicates the minimum of Scr/κ or 1, and max indicates the maximum of Scr/κ or 1. CKD was classified according to the

Kidney Disease: Improving Global Outcomes (KDIGO) 2024 guidelines, utilizing the Cause, GFR category (G1–G5), and Albuminuria category (A1–A3) (CGA) classification system. The GFR categories were defined as: G1: eGFR ≥ 90 ml/min/1.73 m^2; G2: eGFR 60–89 ml/min/1.73 m^2; G3a: eGFR 45–59 ml/min/1.73 m^2; G3b: eGFR 30–44 ml/min/1.73 m^2; G4: eGFR 15–29 ml/min/1.73 m^2; G5: eGFR < 15 ml/min/1.73 m^2. Albuminuria categories based on the urinary albumin-to-creatinine ratio (ACR) were defined as: A1: ACR < 30 mg/g; A2: ACR 30–300 mg/g; A3: ACR > 300 mg/g. CKD was identified in participants who had markers of kidney damage (e.g., ACR ≥ 30 mg/g) and/or decreased eGFR (< 60 ml/min/1.73 m^2), consistent with the KDIGO 2024 guidelines. Participants were staged according to their GFR and albuminuria categories, following the CGA classification system. Because confirmation of persistent kidney dysfunction during at least 3 months required by current guidelines to distinguish acute vs chronic kidney impairment—is not possible using a cross-sectional data set such as NHANES, we compared our CKD estimates with those of the United States Renal Data System 2022 annual report, another epidemiologic analysis of CKD in the US.

Supplementary Results

Baseline characteristics of participants

The final sample size of our study comprised 6,567 participants, with a mean age of 47.27 ± 17.41 years, and 50.07% being male. The weighted prevalence for MASLD was 51.24% (95% CI,

48.9% – 53.6%, n = 3,474), while for NAFLD, it was 48.36% (95% CI, 46.0% – 50.7%, n = 3,333).

Participants diagnosed with MASLD were predominantly male (53.27% vs. 46.73%, P = .002) and older in age (average age: 50.71 vs. 43.66 years, P < .001) compared to those without MASLD.
