# Supplementary material for: Targeting Insulin Resistance and Liver Fibrosis: CKD Screening Priorities in MASLD
Source: Biomedicines. 2025 Apr 1;13(4):842. doi: 10.3390/biomedicines13040842 (PMC12025161; doi:10.3390/biomedicines13040842)
Supplement: Supplementary file 1 [file biomedicines-13-00842-s001.zip › Table S1.pdf]

Table S1. Baseline characteristics of NHANES 2017-2020 individuals with HOMA-IR Data.[a]

Variables	Whole cohort (n = 6567)		HOMA-IR cohort (n = 3280)		MASLD (n = 1728)		Non-MASLD (n = 1552)		*P*-value[b]
Demographics									
Age, *y*	47.27	± 17.41	46.89	± 17.22	50.72	± 16.35	42.78	± 17.18	< .001
Sex									.08
Male, *%*	3298	(50.07)	1646	(51.06)	893	(53.62)	753	(48.31)	
Female, *%*	3269	(49.93)	1634	(48.94)	835	(46.38)	799	(51.69)	
Five cardiometabolic risk factors									
Overweight or Obesity, *%*	5342	(81.61)	2643	(80.49)	1639	(95.21)	1004	(64.72)	< .001
BMI, *kg/m2*	29.47	± 6.89	29.31	± 6.93	32.45	± 6.95	25.95	± 5.08	< .001
Waist circumference, *cm*	99.92	± 16.62	99.43	± 16.60	107.79	± 15.00	90.47	± 13.22	< .001
Hypertension, *%*	1968	(24.50)	965	(23.93)	607	(30.18)	358	(17.23)	< .001
Diastolic blood pressure, *mmHg*	73.83	± 10.74	73.82	± 10.78	75.73	± 10.56	71.77	± 10.62	< .001
Systolic blood pressure, *mmHg*	121.52	± 16.97	120.94	± 16.52	123.41	± 16.30	118.3	± 16.35	< .001
Diabetes, *%*	1016	(11.48)	588	(13.06)	464	(20.66)	124	(4.90)	< .001
Hypertriglyceridemia, *%*	1460	(23.22)	610	(19.92)	478	(29.83)	132	(9.29)	< .001
Low HDL-C, *%*	3241	(48.59)	1610	(48.29)	1074	(61.26)	536	(34.39)	< .001
Lab panel									
Total cholesterol, *mg/dL*	187.37	± 40.53	185.65	± 40.77	189.21	± 42.06	181.84	± 38.99	.009

Triglycerides, *mg/dL*	138.00	(78.00, 162.00)	124.00	(73.00, 148.00)	148.00	(88.00, 170.20)	99.00	(64.00, 116.00)	< .001
HDL-C, *mg/dL*	53.62	± 15.67	53.77	± 15.75	49.45	± 13.65	58.39	± 16.52	< .001
HbA1c, *%*	5.64	± 0.94	5.64	± 0.97	5.86	± 1.17	5.40	± 0.59	< .001
ALT, *U/L*	23.00	(13.00, 27.00)	23.00	(13.00, 26.00)	25.00	(14.00, 28.25)	20.00	(12.00, 23.00)	< .001
AST, *U/L*	21.00	(16.00, 24.00)	21.00	(16.00, 24.00)	21.00	(16.00, 24.00)	21.00	(16.00, 23.00)	.20
ALP, *U/L*	74.96	± 24.30	74.74	± 23.70	77.12	± 23.69	72.19	± 23.46	< .001
GGT, *U/L*	29.00	(14.00, 31.00)	28.00	(15.00, 32.00)	32.00	(17.00, 35.00)	25.00	(13.00, 27.00)	< .001
Albumin, *g/L*	41.23	± 3.22	40.79	± 3.22	40.27	± 3.08	41.35	± 3.29	< .001
Globulin, *g/L*	29.83	± 4.06	30.12	± 4.02	30.33	± 4.07	29.90	± 3.95	.06
Platelet, $10^9/L$	246.60	± 62.40	241.74	± 60.67	244.38	± 63.24	238.91	± 57.67	.10
Total bilirubin, *umol/L*	8.11	(5.13, 10.26)	8.71	(5.13, 10.26)	8.54	(5.13, 10.26)	8.89	(5.13, 10.26)	.30
eGFR, *ml/min/1.73* m^2	93.86	± 21.34	94.84	± 21.12	92.21	± 22.38	97.65	± 20.48	< .001
Creatinine, *mg/dL*	0.88	± 0.34	0.87	± 0.30	0.88	± 0.32	0.86	± 0.28	.03
Urinary ACR, *mg/g*	7.27	(4.73, 13.81)	7.19	(4.68, 13.21)	7.75	(4.95, 14.89)	6.67	(4.48, 11.22)	.002
Noninvasive tests									
Steatosis									
CAP, *dB/m*	263.77	± 62.31	263.71	± 61.05	306.98	± 40.81	217.35	± 42.19	< .001
CAP ≥ 248 dB/m, *%*	3787	(56.97)	1889	(57.89)	1728	(100)	161	(12.76)	< .001
Fibrosis									
LSM, *kPa*	5.70	(4.10, 6.10)	5.60	(4.10, 6.10)	6.20	(4.30, 6.50)	5.00	(3.90, 5.70)	< .001
LSM ≥ 8 kPa, *%*	660	(9.08)	321	(8.70)	239	(12.68)	82	(4.44)	< .001
Insulin resistance, *%*	1594[c]	(44.62)	1594	(44.62)	1184	(63.63)	410	(24.26)	< .001
HOMA-IR[#]	3.95	(1.54, 4.61)	3.95	(1.53, 4.61)	5.53	(2.39, 6.22)	2.25	(1.14, 2.76)	< .001

BMI, body mass index; HDL, high-density lipoprotein; HbA1c, glycosylated hemoglobin; ALT, alanine aminotransferase; AST, aspartate aminotransferase; ALP, Alkaline phosphatase; GGT, gamma-glutamyl transferase; eGFR, estimated glomerular filtration rate; ACR, albumin-to-creatinine ratio; CAP, controlled attenuation parameter; LSM, liver stiffness measure; HOMA-IR: homeostasis model assessment-estimated insulin resistance.

[a]: Method: For categorical variables: “count (weighted percentage)”; for continuous variables with normal distribution: “mean ± standard deviation”; for continuous variables with non-normal distribution: “median (interquartile range)”.

[b]: Continuous variables were compared using t tests and categorical variables using the Rao-Scott chi-squared tests.

[c]: Due to the random selection of participants for fasting blood sampling in each age group, only 3,280 individuals in the study population had computable HOMA-IR values.
