# Supplementary material for: Targeting Insulin Resistance and Liver Fibrosis: CKD Screening Priorities in MASLD
Source: Biomedicines. 2025 Apr 1;13(4):842. doi: 10.3390/biomedicines13040842 (PMC12025161; doi:10.3390/biomedicines13040842)
Supplement: Supplementary file 1 [file biomedicines-13-00842-s001.zip › Table S2.pdf]

Table S2. Weighted logistic regression analyses of the association between all variables and CKD Prevalence.

		GFR category ≥ G1			GFR category ≥ G3		
Model	variables	OR (95%CI)		*P* –value	OR (95%CI)		*P* –value
Adjustment Model							
Age and sex	Age	1.32	(1.07 – 1.61)	0.01	1.36	(1.14 – 1.39)	＜0.001
	Male	1.26	(1.12 – 1.59)	0.020	1.18	(0.98 – 1.46)	0.08
	Masld	1.32	(1.07 – 1.61)	0.01	1.34	(0.99 – 1.82)	0.06
Model *1*	Age	1.05	(1.04 – 1.05)	＜0.001	1.12	(1.10 – 1.14)	＜0.001
	Male	1.24	(1.00 – 1.55)	0.050	1.16	(0.93 – 1.43)	0.18
	Masld	1.29	(1.05 –1.59	0.02	1.34	(0.99 – 1.32)	0.06
	Hypertension	1.67	(1.33 – 2.08)	＜0.001	0.93	(0.65 – 1.32)	0.66
Model *2*	Age	1.05	(1.05 – 1.06)	＜0.001	1.12	(1.10 – 1.14)	＜0.001
	Male	1.24	(0.99 – 1.54)	0.06	1.10	(0.89 – 1.35)	0.35
	Masld	1.30	(1.02 – 1.65)	0.04	1.22	(0.88 – 1.70)	0.22
	Overweight or Obesity	1.06	(0.72 – 1.57)	0.75	1.75	(1.16 – 2.64)	0.01
Model *3*	Age	1.05	(1.05 – 1.06)	＜0.001	1.12	(1.10 – 1.14)	＜0.001
	Male	1.30	(1.05 – 1.61)	0.02	1.21	(0.97 – 1.51)	0.09
	Masld	1.19	(0.98 – 1.45)	0.07	1.18	(0.86 – 1.61)	0.29
	Hypertriglyceridemia	1.54	(1.21 – 1.97)	0.001	1.70	(1.22 – 2.37)	0.003
Model *4*	Age	1.05	(1.05 – 1.06)	＜0.001	1.12	(1.10 – 1.14)	＜0.001
	Male	1.45	(1.18 – 1.78)	0.001	1.38	(1.09 – 1.76)	0.01
	Masld	1.15	(0.93 – 1.42)	0.20	1.15	(0.84 – 1.58)	0.37
	Low HDL-C	1.65	(1.37 – 1.99)	＜0.001	1.79	(1.36 – 2.36)	＜0.001
Model *5*	Age	1.04	(1.04 – 1.05)	＜0.001	1.12	(1.10 – 1.14)	＜0.001
	Male	1.37	(1.10 – 1.71)	0.007	1.20	(0.96 – 1.51)	0.11
	Masld	1.06	(0.86 – 1.31)	0.54	1.23	(0.89 – 1.70)	0.20
	Diabetes	3.48	(2.87 – 4.21)	< 0.001	1.53	(1.11 – 2.11)	0.01
Model *6*	Age	1.05	(1.04 – 1.05)	＜0.001	1.12	(1.10 – 1.14)	＜0.001
	Male	1.47	(1.17 – 1.83)	0.002	1.32	(1.04 – 1.67)	0.02
	Masld	1.32	(1.03 – 1.69)	0.03	1.01	(0.72 – 1.43)	0.94
	Hypertension	1.67	(1.33 – 2.09)	＜0.001	0.93	(0.65 – 1.33)	0.66
	Overweight or Obesity	0.95	(0.63 – 1.43)	0.79	1.58	(1.04 – 2.40)	0.03
	Hypertriglyceridemia	1.35	(1.05 – 1.75)	0.02	1.42	(0.98 – 2.07)	0.06
	Low HDL-C	1.51	(1.23 – 1.85)	＜0.001	1.57	(1.15 – 2.13)	0.006
Model *7*	Age	1.04	(1.04 –1.05)	＜0.001	1.12	(1.10 – 1.14)	＜0.001
	Male	1.52	(1.21 – 1.92)	0.001	1.34	(1.05 – 1.70)	0.02
	Masld	0.94	(0.65 – 1.47)	0.74	0.97	(0.68 – 1.39)	0.86
	Hypertension	1.63	(1.30 – 2.04)	＜0.001	0.92	(0.64 – 1.32)	0.64
	Overweight or Obesity	0.97	(0.64 – 1.45)	0.87	1.58	(1.03 – 2.41)	0.04
	Hypertriglyceridemia	1.20	(0.93 – 1.56)	0.15	1.38	(0.96 – 1.99)	0.08
	Low HDL-C	1.35	(1.11 – 1.64)	0.005	1.52	(1.10 – 2.09)	0.01
	Diabetes	3.12	(2.57 – 3.78)	＜0.001	1.32	(1.12 – 1.83)	0.01
Model *8*	Age	1.05	(1.04 – 1.06)	＜0.001	1.11	(1.09 – 1.14)	＜0.001

Male	0.99	(0.65 – 1.51)	0.95	1.30	(0.75 – 2.27)	0.32
Masld	0.87	(0.46 – 1.63)	0.64	0.85	(0.40 – 1.82)	0.65
Hypertension	1.58	(1.01 – 1.83)	0.05	0.53	(0.36 – 0.79)	0.003
Overweight or Obesity	1.74	(0.89 – 3.40)	0.10	1.51	(0.67 – 3.39)	0.30
Hypertriglyceridemia	0.97	(0.61 – 1.54)	0.89	0.89	(0.53 – 1.50)	0.64
Low HDL-C	1.52	(1.00 – 2.32)	0.05	1.69	(0.97 – 2.94)	0.06
Alcohol drinking	0.99	(0.69 – 1.40)	0.94	1.11	(0.74 – 1.67)	0.58
Diabetes	3.06	(2.52 – 3.68)	＜0.001	1.24	(1.07 – 1.72)	0.03
Antihypertensive medication use	1.42	(1.12 – 1.81)	0.006	0.44	(1.80 – 1.07)	0.07
Smoking	0.81	(0.35 – 1.83)	0.58	1.09	(0.77 – 1.55)	0.59

MASLD, metabolic dysfunction-associated steatotic liver disease; BMI, body mass index; HDL, high-density lipoprotein; HbA1c, glycosylated hemoglobin; CAP, controlled attenuation parameter; LSM, liver stiffness measure, CI, confidence interval; OR, odds ratio.

Model 1: Adjusted for age, sex. and hypertension.

Model 2: Adjusted for age, sex. and overweight or obesity.

Model 3: Adjusted for age, sex and hypertriglyceridemia.

Model 4: Adjusted for age, sex and Low HDL-C.

Model 5: Adjusted for age, sex and Diabetes.

Model 6: Adjusted for age, sex, hypertension, overweight/obesity, hypertriglyceridemia, and low HDL-C.

Model 7: Further adjusted for diabetes based on Model 6.

Model 8: Further adjusted for smoking, alcohol consumption, and antihypertensive medication use based on Model 7.
