# Supplementary material for: Targeting Insulin Resistance and Liver Fibrosis: CKD Screening Priorities in MASLD
Source: Biomedicines. 2025 Apr 1;13(4):842. doi: 10.3390/biomedicines13040842 (PMC12025161; doi:10.3390/biomedicines13040842)
Supplement: Supplementary file 1 [file biomedicines-13-00842-s001.zip › Table S3.pdf]

Table S3. Interaction Effects of MASLD with Key Variables: Odds Ratios and Confidence Intervals.

Model	variables	OR (95%CI)		P –value
Adjustment Model				
*Model 9 + Age *MASLD*	Age *MASLD	1.01	(0.99 –1.03)	0.18
*Model 9 + Sex *MASLD*	Sex *MASLD	0.53	(0.33 –0.87)	0.01
*Model 9 + Hypertension *MASLD*	Hypertension *MASLD	1.08	(0.65 –1.81)	0.75
*Model 9 + Overweight or Obesity *MASLD*	Overweight or Obesity *MASLD	1.87	(0.86 –4.05)	0.11
*Model 9 + Diabetes *MASLD*	Diabetes *MASLD	0.68	(0.31 –1.49)	0.32
*Model 9 + Low HDL-C *MASLD*	Low HDL-C *MASLD	1.45	(0.76 –2.77)	0.25
*Model 9 + Hypertriglyceridemia *MASLD*	Hypertriglyceridemia *MASLD	1.92	(0.90 –4.08)	0.09
*Model 9 + Insulin resistance *MASLD*	Insulin resistance *MASLD	2.02	(1.05 - 3.89)	0.04

MASLD, metabolic dysfunction-associated steatotic liver disease; CI, confidence interval; OR, odds ratio. HOMA-IR: homeostasis model assessment-estimated insulin resistance.

Model 9: Further adjusted for insulin resistance based on Model 8

Model 9 + Age *MASLD: Introduces a multiplicative interaction term between Age and MASLD in Model 9.

Model 9 + Sex *MASLD: Introduces a multiplicative interaction term between Sex and MASLD in Model 9.

Model 9 + Hypertension *MASLD: Introduces a multiplicative interaction term between Hypertension and MASLD in Model 9.

Model 9 + Overweight or Obesity *MASLD: Introduces a multiplicative interaction term between Overweight or Obesity and MASLD in Model 9.

Model 9 + Diabetes *MASLD: Introduces a multiplicative interaction term between Diabetes and MASLD in Model 9.

Model 9 + Low HDL-C *MASLD: Introduces a multiplicative interaction term between Low HDL-C and MASLD in Model 9.

Model 9 + Hypertriglyceridemia *MASLD: Introduces a multiplicative interaction term between Hypertriglyceridemia and MASLD in Model 9.

Model 9 + Insulin resistance *MASLD: Introduces a multiplicative interaction term between insulin resistance and MASLD in Model 9.
